# Replication of Host *St6galnac1* -Microbiome Association Across Distinct Experimental Conditions in Heterogeneous Stock Rats

**DOI:** 10.64898/2026.10.09.758003

**Published:** 2026-10-16

**Authors:** Mohammad Sadegi, Benjamin B Johnson, Thiago Missfeldt Sanches, Gracie Ang, Jennifer Cao, Madison Ambre, Molly Brennan, Olivier George, Rob Knight, Amelie Baud, Abraham A. Palmer, Oksana Polesskaya

## Abstract

The gut microbiome is shaped by both environmental and host genetic factors. Identifying specific host loci is essential for understanding the heritability of microbiome-associated traits. The gene *St6galnac1* encodes a sialyltransferase involved in mucin O-glycosylation. Tonnelé et al. (2025) identified significant associations between a locus containing *St6galnac1* gene, and the abundance of genus *Paraprevotella* using cecal samples collected from fasted animals. This association was replicated across three cohorts, and paralleled with association found in humans between a functionally related sialyltransferase gene *ST6GAL1* and *Paraprevotella* .

Given the sensitivity of microbiome analyses to factors including sampling site, feeding status, and analytical framework, independent replication of these findings is important and it is also important to know if the association generalizes across other conditions or is highly specific to certain environments. Here, we performed a replication analysis using stool (not cecal) samples from 174 HS rats that were not fasted prior to collection. Stool samples underwent 16S rRNA gene sequencing, and microbial community structure was analyzed using compositionally aware methods. The rats were genotyped at the top SNP that was associated with *Paraprevotella* in the Tonnelé et al. (2025) study. The association between genotype and *Paraprevotella* abundance was tested using a linear mixed model accounting for sex, cohort, and genetic relatedness. We successfully replicated (p=0.015) *St6galnac1 - Paraprevotella* association.

These findings support the robustness of the *St6galnac1 - Paraprevotella* association across different sampling strategies and experimental conditions.

